# Supplementary material for: Complexome profiling of the Chlamydomonas psb28 mutant reveals TEF5 as an early PSII assembly factor
Source: Plant Cell. 2025 Mar 17;37(6):koaf055. doi: 10.1093/plcell/koaf055 (PMC12164590; doi:10.1093/plcell/koaf055)
Supplement: koaf055_Supplementary_Data [file koaf055_supplementary_data.zip › psb28_interactive.zip!/plots/intensity/qualityPlots/peptides/_Cre08.g358538.t1.1.html]

### Cre08.g358538.t1.1

Trivial name:   
SeqCoverage [median]: 18.50  
Mass: 63.87 kDa  
  
 

| Sequence | MissedCleavage | Mass [Da] | wtIntensity | muIntensity | Score |
| --- | --- | --- | --- | --- | --- |
| AANTALPTFLYAMPFTK | 0 | 1855.95 | 1397.03 | 0.00 | 85.42 |
| AFSTSNTK | 0 | 854.41 | 688.67 | 2089.67 | 108.46 |
| DDHTQAPGLEAMR | 0 | 1439.65 | 238.56 | 0.00 | 46.07 |
| GSLVLDATGHSR | 0 | 1211.63 | 0.00 | 3688.50 | 94.19 |
| KDAVDAAAR | 1 | 915.48 | 346.50 | 469.39 | 64.84 |
| LNLPQIR | 0 | 852.52 | 0.00 | 1246.96 | 77.75 |
| MMGAAPTVADTIVDQLSRPADK | 1 | 2286.13 | 1981.05 | 7144.49 | 83.78 |
| VLAIGGTAGMVHPSTGFMISR | 0 | 2101.08 | 5579.37 | 14827.79 | 85.81 |
|  |  |  | 10231.2 | 29466.8 |  |
